# Assessment of vitreous haze using ultra-wide field retinal imaging

**DOI:** 10.1186/s12348-016-0105-0

**Published:** 2016-09-29

**Authors:** Drew Dickson, Aniruddha Agarwal, Mohammad Ali Sadiq, Muhammad Hassan, Robin High, Quan Dong Nguyen, Yasir J. Sepah

**Affiliations:** 1 Ocular Imaging Research and Reading Center (OIRRC), Omaha, NE 68198-5540 USA; 2 College of Public Health, University of Nebraska Medical Center, 984355 Medical Center, Omaha, NE USA; 3 Advanced Eye Center, Department of Ophthalmology, Post Graduate Institute of Medical Education and Research (PGIMER), Chandigarh, India

**Keywords:** Vitreous haze, Scanning laser ophthalmoscopy, Ultra-wide field, Fundus imaging, Uveitis

## Abstract

**Background:**

Conventional fundus imaging has been used to assess vitreous haze (VH) in patients with uveitis. Ultra-wide field (UWF) retinal imaging that uses scanning laser technology has not been evaluated for the detection of VH. This pilot study evaluates the ability of UWF imaging in detecting VH.

Patients with intermediate, posterior, or panuveitis were examined to assess the level of VH using slit-lamp biomicroscopy. Colored fundus images were acquired using a Carl Zeiss FF450 camera. The same photographer obtained fundus images of the same eyes during the same visit by Optos UWF P200Tx retinal camera. Two graders independently analyzed UWF fundus images for presence or absence of VH, without quantifying the degree of VH using any scale. The images were analyzed using the composite red plus red-free wavelengths utilized by the Optos UWF camera and by using each wavelength exclusively. These findings were compared to clinical detection of VH and detection of VH using conventional fundus photography.

**Results:**

Ninety-two eyes were included in the study. For composite UWF images, sensitivity was 0.27, specificity was 0.88, PPV was 0.31, NPV was 0.86, positive LR was 2.25, and negative LR was 0.83. For the conventional Zeiss images, sensitivity was 0.5, specificity was 0.84, PPV was 0.33, NPV was 0.91, positive LR was 3.13, and negative LR was 0.6.

Agreement between the composite UWF and Zeiss techniques was substantial with *k* = 0.64. Inter-observer agreement for composite UWF images was also substantial with *k* = 0.65. Inter-observer agreement for Zeiss images was moderate with *k* = 0.471. Intra-observer agreement for both imaging modalities was substantial with a composite UWF *k* = 0.76 and Zeiss *k* = 0.7.

**Conclusions:**

UWF fundus imaging using scanning laser technique may be used to assess VH and employed in the management of intermediate, posterior, and panuveitis.

## Background

Uveitis is a group of inflammatory ocular conditions that may cause significant visual impairment. Recent estimates suggest that uveitis causes between 5 and 20 % of legal blindness in the USA and Europe and up to 25 % of blindness in the developing world [1]. Protein exudates and inflammatory cells in yes with uveitis distort a clear view of the fundus and cause vitreous haze (VH) [2]. Therefore, detecting and grading vitreous haze is important for patient management and has been approved by the United States Food and Drug Administration (US-FDA) as an outcome measure for clinical trials in uveitis [3–7].

Fundus imaging using conventional color photographs is commonly used to assess VH in patients with uveitis. A five-step scale of VH based upon ophthalmoscopic clarity of the fundus was proposed by Kimura and associates in 1959 [8]. A six-step scale of VH was created at the National Eye Institute (NEI) in 1985 [9]. This scale was approved by the Standardization of Uveitis Nomenclature (SUN) Working Group in 2005 as an acceptable method of grading VH in clinical research [10]. A nine-step ordinal scale was recently designed by Davis et al. for grading VH [11].

While VH grading of conventional fundus photographs has been validated and standardized, it only provides a 30° field of view. Ultra-wide field (UWF) retinal imaging using scanning laser technology provides a field of view of approximately 200°, which covers 82 % of the retina in a single image [12, 13]. Due to the superior ability of UWF imaging in visualizing the peripheral retina, it has been reported to aid in the management of several posterior segment disorders including uveitis [13–17]. A prospective study by Campbell et al. suggested that using UWF imaging and fluorescein angiography (FA) might alter the clinical management of patients with posterior non-infectious uveitis [14].

UWF imaging has not been evaluated for the detection of VH due to the belief that lasers can penetrate media opacities such as cataracts, vitreous hemorrhage, and inflammation and therefore cannot effectively measure VH [18]. However, given the increasing application of UWF imaging and its ability to aid in the management of patients with uveitis, it is important to explore the ability of UWF imaging to identify vitreous haze. This prospective pilot study aims to determine if UWF imaging is able to identify vitreous haze.

## Methods

### Image acquisition and analysis

Patients diagnosed with intermediate, posterior, or panuveitis at a tertiary eye care center were examined with slit-lamp biomicroscopy to assess the level of VH using the NEI scale. Since the study aimed at determining the ability of UWF imaging in detecting VH, patients with no or only mild VH (≤2+ VH) were included in the study. Colored fundus images (30°, standard 2 M field) were acquired during each patient visit using Carl Zeiss FF450N (Carl Zeiss Meditech, Dublin, CA) camera. An Optos UWF 200° retinal camera (Optos P200Tx, Optos, Scotland, UK) that uses green (532 nm) and red (633 nm) lasers was also used to obtain fundus images of the same eyes during the same patient visit by a single photographer. The eyes with significant cataract, hemorrhage, corneal opacity, and poor cooperation for photography were excluded.

Two graders were masked to the clinical data and independently analyzed UWF fundus images on a single computer monitor for presence or absence of VH, without quantifying the degree of VH using any scale. They did this for composite red plus red-free images and for exclusively red or red-free images. The graders then analyzed the conventional fundus images obtained from the Zeiss camera for the presence or absence of VH, again without quantifying the degree of VH using any scale. These images were analyzed again in the same manner by the first grader 1 month later. A third grader, masked to the clinical data, independently analyzed the UWF and Zeiss images the first two graders disagreed upon to serve as a tie-breaker in order to perform the statistical evaluation. The findings were then compared to the gold standard, i.e., clinical examination, to measure the ability of the UWF technique to detect VH.

### Statistical evaluation

Sensitivity, specificity, positive and negative predictive values (PPV and NPV), and positive and negative likelihood ratios (LR) for both the UWF and Zeiss imaging techniques in detecting VH were calculated. An agreement between the two imaging techniques, inter-observer agreement, and intra-observer agreement in detecting VH were calculated using the weighted kappa statistic to account for any random chance in agreement. An adjusted inter-observer agreement was also determined by awarding agreement between the two graders for all images that had a clinical grade of 0 or 0.5+ VH. A 95 % confidence interval was calculated for each kappa value. The kappa measure lies on a scale from −1 to 1, where negative values represent agreement less than chance, 0 represents agreement no more than chance, and 1 represents perfect agreement [19]. A commonly used scale designates a *k* value of 0.01–0.20 as slight agreement, 0.21–0.40 as fair agreement, 0.41–0.60 as moderate agreement, 0.61–0.80 as substantial agreement, and 0.81–0.99 as almost perfect agreement [20].

## Results

Images from 92 eyes (47 patients) were analyzed. Six Zeiss images were not of sufficient quality and were not used in the kappa calculations of intra- and inter-observer agreement of Zeiss image analysis or agreement between the UWF and Zeiss images. All of the non-gradable images had no clinical VH. All of the composite UWF images and red-free images were gradable, while three of the red-only images were not of sufficient quality and disregarded for the calculations. One of these three red-only images had clinical VH. Eighty of the 92 eyes had no clinically detectable VH. Five eyes had 0.5+ VH, four eyes had 1+ VH, one eye had 1.5+ VH, and two eyes had 2+ VH on clinical examination using the NEI scale. None of the eyes included in the analysis had grade 3+ or 4+ VH. The mean clinical VH of the eyes with active uveitis was 1+ on the NEI scale.

**Fig. 1:**
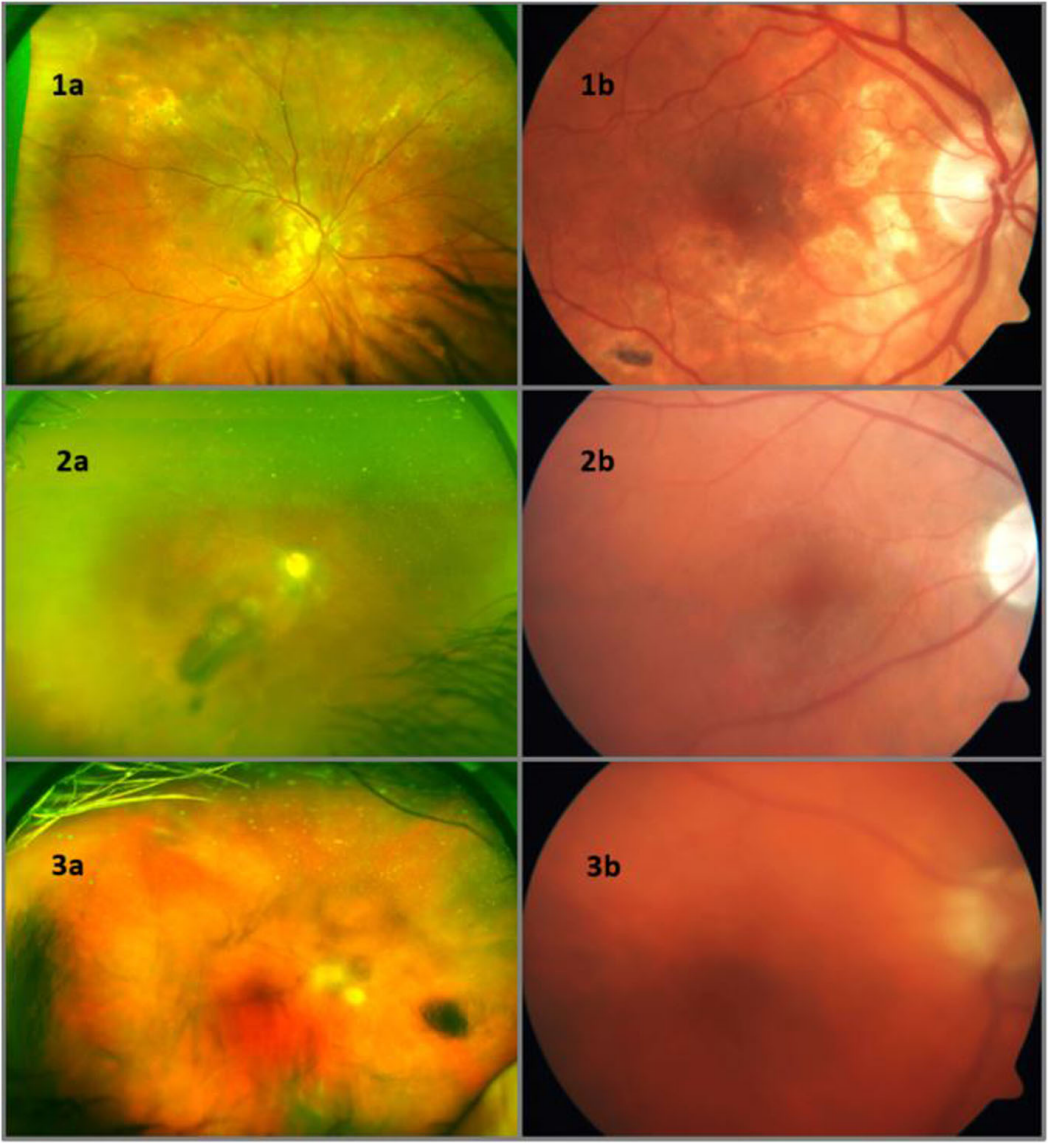
**1a** Composite UWF image of no clinical VH. **1b** FF450N image of no clinical VH. **2a** Composite UWF image of clinical 1+ VH. **2b** FF450N image of clinical 1+ VH. **3a** Composite UWF image of clinical 2+ VH. **3b** FF450N image of clinical 2+ VH. Clinical grading of VH was based on the NEI scale [9]. Composite UWF and FF450N images were obtained from the same eye for each respective grade of VH

**Fig. 2:**
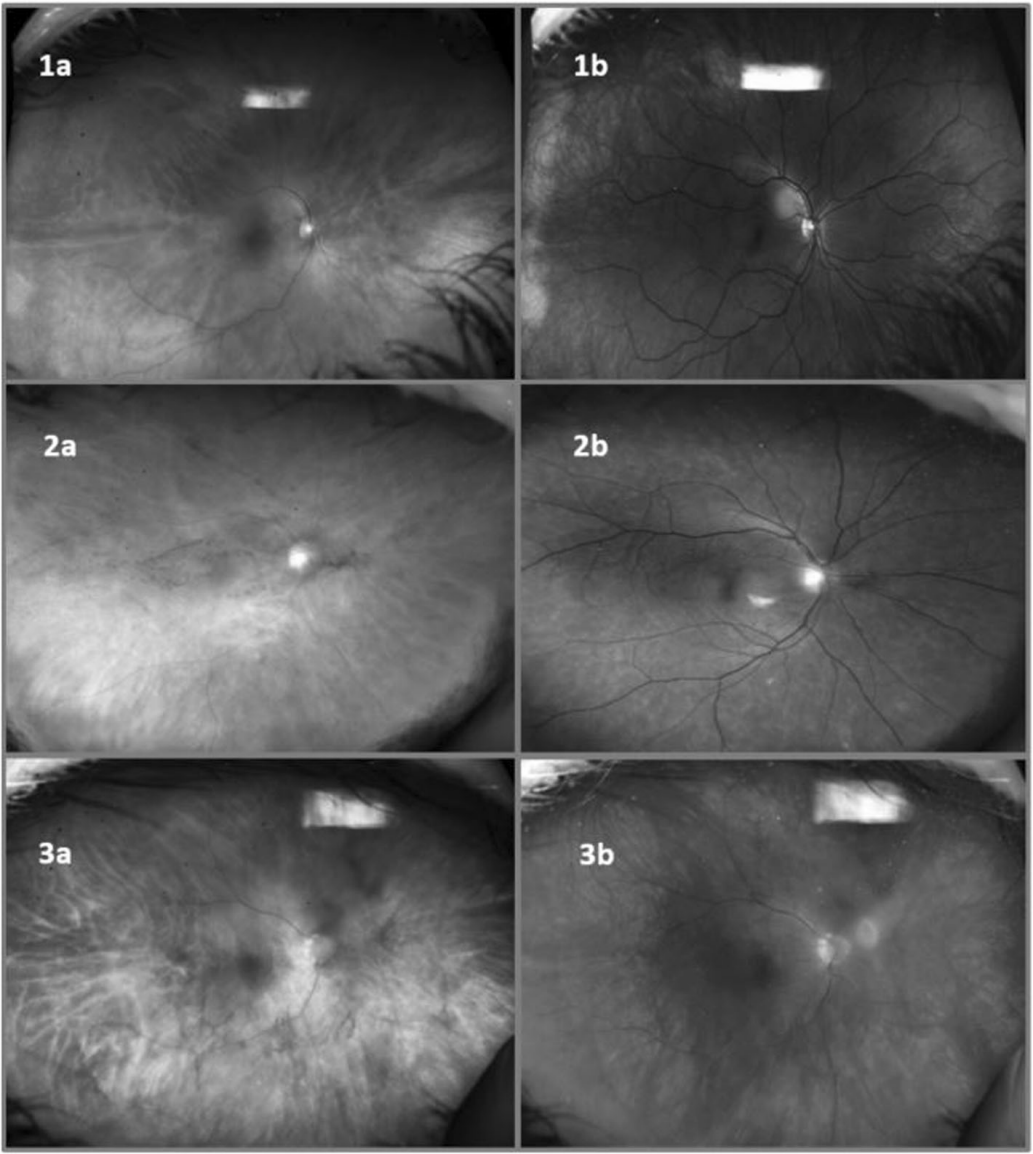
**1a** Red-only UWF image of no clinical VH. **1b** Red-free UWF image of no clinical VH. **2a** Red-only UWF image of clinical 1+ VH. **2b** Red-free UWF image of 1+ clinical VH. **3a** Red-only UWF image of clinical 2+ VH. **3b** Red-free UWF image of 2+ clinical VH

In comparing the UWF imaging and the conventional Zeiss photographs to the clinical VH assessment, the sensitivity was 0.27 and 0.5 for Optos UWF and conventional photographs, respectively. Specificity was 0.88 for Optos UWF and 0.84 for conventional photographs. The PPV was 0.31 and 0.33, while the NPV was 0.86 and 0.91 for Optos UWF and conventional photographs, respectively. The positive LR was 2.25 and the negative LR was 0.83 for Optos UWF, whereas the positive LR was 3.13 and the negative LR was 0.6 for conventional photographs. For the red-only UWF images, the sensitivity was 0.50, specificity was 0.853, PPV was 0.353, NPV was 0.914, positive LR was 3.4, and negative LR was 0.531. For the red-free UWF images, the sensitivity was 0.417, specificity was 0.857, PPV was 0.313, NPV was 0.904, positive LR was 2.92, and negative LR was 0.68. These results are summarized in Table 1. Figure 1 provides comparison of the composite Optos UWF and Zeiss techniques in the eyes with and without VH, while Fig. 2 provides comparison between the red-only Optos UWF and red-free Optos UWF imaging techniques in the eyes with and without VH.

**Table 1:** Statistical evaluation of vitreous haze detection (clinical haze present in 12 eyes)

Statistical measure	Composite UWF images (haze + in 9 eyes)	Red-only UWF images (haze + in 17 eyes)	Red-free UWF images (haze + in 16 eyes)	Conventional Zeiss images (haze + in 16 eyes)
Sensitivity	0.27	0.50	0.42	0.50
Specificity	0.88	0.85	0.86	0.84
PPV	0.31	0.35	0.31	0.33
NPV	0.86	0.91	0.90	0.91
Positive LR	2.25	3.40	2.92	3.13
Negative LR	0.83	0.53	0.68	0.60

*UWF* ultra-wide field imaging, *PPV* positive predictive value, *NPV* negative predictive value, *LR* likelihood ratio

The agreement between the composite UWF and Zeiss techniques was substantial with the agreement on 76/86 eyes (88.4 %) and *k* = 0.64 (95 % CI 0.44–0.84). The agreement between the composite UWF and red-only UWF images was moderate with the agreement on 75/87 eyes (86.2 %) and *k* = 0.49 (95 % CI 0.25–0.74). Substantial agreement was observed between the composite UWF and red-free UWF images in detecting VH with agreement on 80/88 eyes (90.9 %) and *k* = 0.64 (95 % CI 0.41–0.87). There was substantial agreement between the two observers for detection of VH using composite Optos UWF images with agreement on 83/92 eyes (90.2 %) and a *k* = 0.65 (95 % CI 0.45–0.86). Red-only inter-observer agreement was substantial with agreement on 78/86 eyes (90.7 %) and *k* = 0.66 (95 % CI 0.45–0.88). Red-free inter-observer agreement was moderate with agreement on 79/90 eyes (87.8 %) with *k* = 0.58 (95 % CI 0.36–0.79). For the Zeiss technique, there was agreement on 67/86 eyes (77.9 %) and a modest *k* = 0.47 (95 % CI 0.27–0.67).

**Fig. 3:**
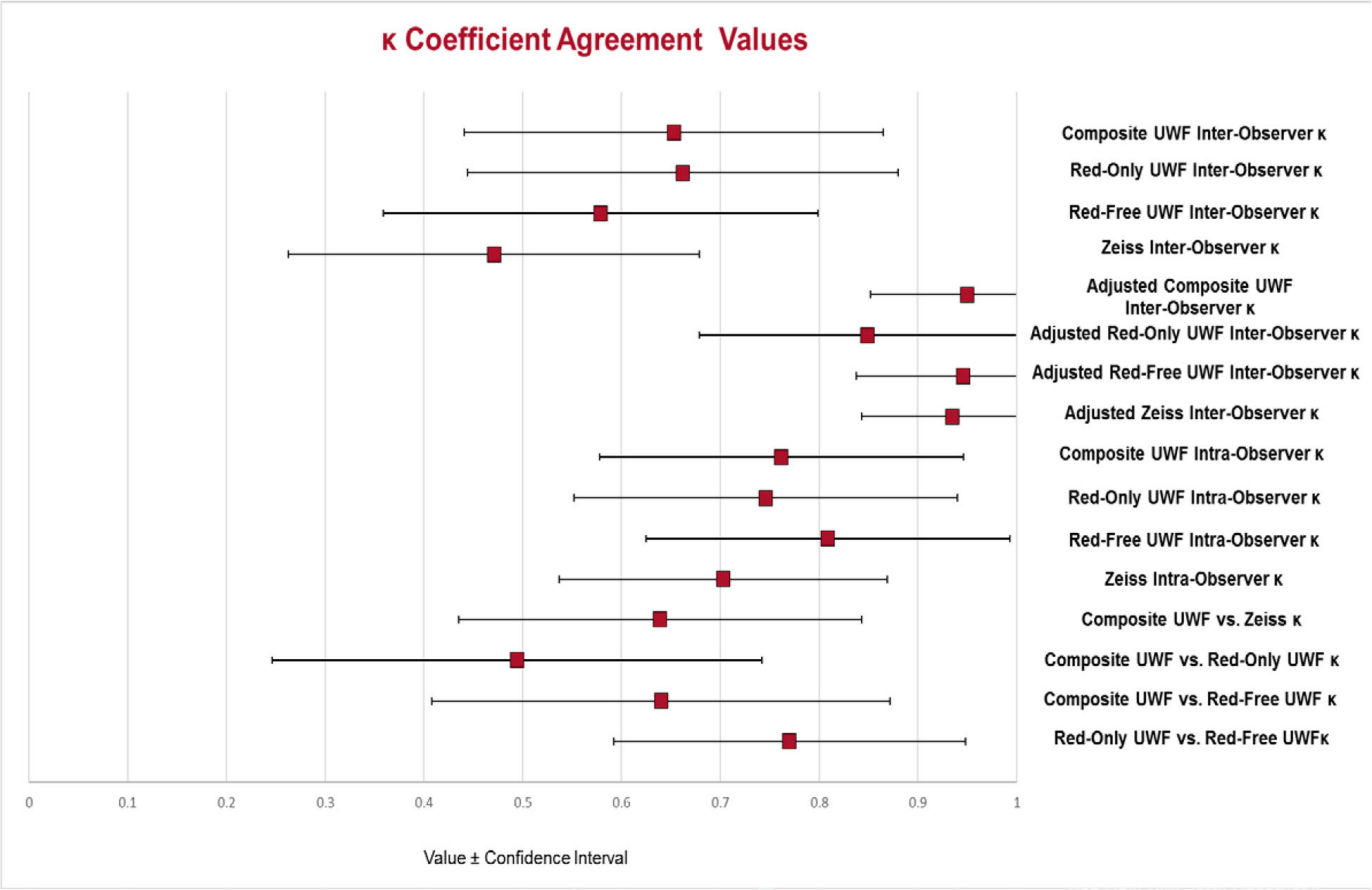
Ultra-wide field (UWF) and Zeiss *k* coefficient agreement values. Inter-observer agreement, intra-observer agreement, and agreement between the graders and imaging techniques are reported by the *red squares* with their confidence intervals

There was near-perfect agreement for all imaging modalities using the adjusted inter-observer agreement. Agreement occurred on 91/92 eyes (97.8 %) with a *k* = 0.95 (95 % CI 0.85–1.0) for the composite UWF technique. For the red-only UWF images, agreement occurred on 83/86 eyes (96.5 %) with a *k* = 0.85, and for the red-free UWF images, agreement occurred on 89/90 eyes (98.9 %) with a *k* = 0.95. Likewise, the Zeiss technique had near-perfect agreement for the adjusted inter-observer agreement with agreement on 84/86 eyes (97.7 %) with a *k* = 0.94 (95 % CI 0.85–1.0). Intra-observer agreement for the composite UWF images was substantial with agreement on 86/92 eyes (93.5 %) and *k* = 0.76. Similarly, substantial intra-observer agreement was observed for the red-only UWF images with agreement on 80/86 eyes (93.0 %) and *k* = 0.75 (95 % CI 0.56–0.94). The red-free UWF intra-observer agreement was near-perfect with agreement on 86/90 eyes (95.6 %) and *k* = 0.81 (95 % CI 0.63–0.99). Intra-observer agreement for conventional images was substantial with agreement on 77/88 eyes (87.5 %) and *k* = 0.70 (Fig. 3).

## Discussion

Detecting and grading VH is important for both patient care and as an outcome measure in clinical trials for patients with uveitis. The NEI scale and new nine-step scale developed by Davis et al. have demonstrated adequate ability to detect and grade VH in a reproducible manner, but with some limitations. Likewise, the results of this study indicate that UWF imaging may be used to assess VH and be employed in the management of patients with uveitis with some limitations as well.

The results of this pilot study suggest that UWF using SLO does indeed detect VH similar to conventional imaging techniques despite the belief that SLO may pierce through opacities caused by inflammation [18]. Similar results were observed in a recent study by Keane et al. in which spectral domain optical coherence tomography (SD-OCT), which also uses scanning laser technology, was used to assess and quantify VH based on the reflectivity pattern [21]. Keane et al. demonstrated that SD-OCT may be used as an objective measure to quantify VH [21]. In SLO, the emitted laser wavefront is composed of coherent light (spatial and temporal) allowing it to be focused at a tight spot and cover great distances. Properties of diffraction physics apply to laser waves much like visible light. Thus, protein exudates and inflammatory cells may scatter the incident laser beams albeit with a different pattern, resulting in detection of VH. Although both the NEI and Davis scales have shown to provide sufficient reproducibility, the growing use and benefits of UWF imaging in the management of patients with uveitis makes it important to evaluate its ability to detect VH. This study did not intend to develop a scale for UWF grading of VH since it first needs to be determined if UWF can detect VH. Sensitivity, specificity, PPV, NPV, and positive and negative LR using composite, red-only, and red-free UWF imaging were similar to the values seen with conventional imaging (Table 1). It is worth emphasizing here that all UWF images and conventional photographs demonstrated poor sensitivity in detecting haze but good specificity (Table 1).

**Fig. 4:**
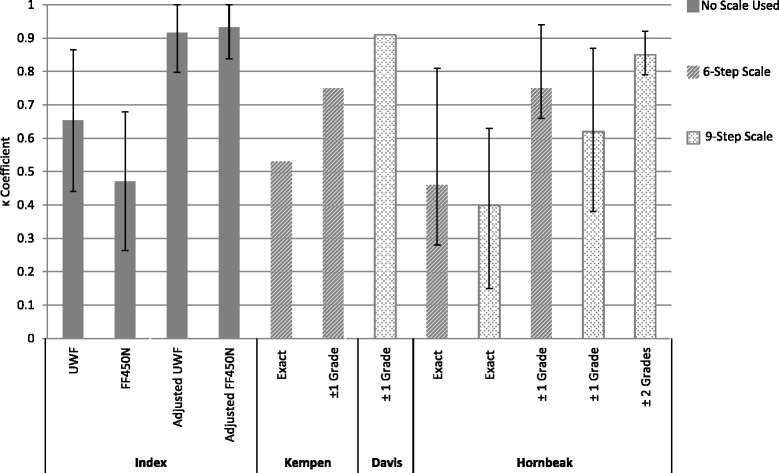
*k* coefficient agreement values of inter-observer agreement in different studies assessing vitreous haze (VH). Graphic representations of the agreement between the graders of the index study, Kempen study [23], Davis study [11], and Hornbeak study [22] are shown. The *k* values are reported by the top of the *gray bars* with their confidence intervals for the current study. No confidence interval was reported for the average *k* in the Kempen or Davis studies, and the Hornbeak intervals represent the range for the average inter-observer *k* value. *6-step scale* NEI scale, *9-step scale* Davis scale

The inter-observer agreement with composite UWF imaging (*k* = 0.65) was substantial, while inter-observer agreement with Zeiss (*k* = 0.47) was moderate. Additionally, both the red-only UWF (*k* = 0.66) and red-free UWF (*k* = 0.58) inter-observer agreements were higher than the Zeiss inter-observer agreement. This suggests that UWF imaging may provide a less ambiguous assessment of VH than conventional imaging. When compared to the composite UWF image analysis, the red-only and red-free UWF images did not differ much in terms of their reproducibility. Red-only images had better inter-observer agreement, while red-only images had better intra-observer agreement. A comparison of the inter-observer agreements for the composite UWF and Zeiss images in this study with inter-observer agreements in the Kempen, Davis, and Hornbeak studies can be seen in Fig. 4.

It should be noted that the calculation of the kappa statistic for this study differed from the calculation of kappa in the Davis study. For this study, kappa was calculated based on the graders’ analysis of haze being present or absent and not on haze gradation. In the Davis study, kappa was determined by giving credit for both exact inter-observer agreement and inter-observer agreement within one haze grade [11]. In the index study, if one grader thought there was no VH (0 VH in the Davis scale) and the other thought there was slight VH (1+ VH on the Davis scale), no credit would be given for agreement. This accounts for the discrepancy in the kappa values for inter-observer agreement of conventional images in this study (*k* = 0.47) versus the average kappa value for inter-observer agreement in the Davis study (*k* = 0.91). Although kappa was not calculated for exact inter-observer agreement in the Davis study, the exact agreement between the graders only occurred 48 ± 7.6 % of the time [11]. Exact agreement occurred on 77.9 % of images for this study. In the Hornbeak study, kappa was reported for exact inter-observer agreement for the six- and nine-step scales, with an average *k* = 0.46 and 0.40, respectively [22]. This is similar to the inter-observer agreement for conventional images seen in this study (*k* = 0.47). The adjusted inter-observer agreement calculated for this study, which awarded agreement for images that had clinical grade of 0 or 0.5+ VH, represents a kappa calculation method more similar to that used by Davis et al. This adjusted agreement had a *k* value for both the UWF and Zeiss imaging modalities (*k* = 0.95 and 0.94, respectively) that was much closer to the *k* value (*k* = 0.91) for the average inter-observer agreement in the Davis study [11].

It is relevant to note that while a substantial agreement has been found between conventional and UWF imaging in detecting VH, these imaging modalities cannot be used interchangeably during the follow-up imaging of patients with uveitis. In addition, visibility of the retinal nerve fiber layer (RNFL) and the posterior pole is an important parameter in detecting and grading VH in the eyes with uveitis. Compared to UWF imaging, conventional imaging may provide images with better detailing of the posterior pole and RNFL.

While UWF imaging appears to be effective at detecting VH, this study has limitations. This study was performed at a single tertiary care center, which may cause sampling bias. The results of this study also need to be validated. Furthermore, quantification of haze was not performed. Although UWF imaging appears to be useful in detecting VH, it remains unknown if UWF imaging can adequately allow for VH grading. The results of the index study cannot provide details of the ability of UWF imaging in quantifying VH among patients with uveitis. The majority of the disagreement between the graders occurred on the eyes that either had no clinical haze present or mild VH, indicating that UWF imaging may be poor at detecting low levels of VH. Additionally, UWF imaging needs to be validated for detecting haze in the presence of cataracts, hemorrhage, and other corneal opacities. Future studies will need to assess the ability of UWF imaging in accurately and reproducibly grading VH.

## Conclusions

The results of this study suggest that UWF imaging may be used to detect VH. With the growing use and benefits of UWF imaging, the ability to assess VH using UWF images may help management of patients with uveitis more efficient and effective.

## Abbreviations

FA: Fluorescein angiography
LR: Likelihood ratio
NEI: National Eye Institute
NPV: Negative predictive value
PPV: Positive predictive value
SLO: Scanning laser ophthalmoscopy
SUN: Standardization of Uveitis Nomenclature
UWF: Ultra-wide field
VH: Vitreous haze
